# MRI of pediatric ovarian masses: validation of the O-RADS framework

**DOI:** 10.1007/s00330-025-11444-0

**Published:** 2025-02-13

**Authors:** Alice M. Munari, Caterina B. Monti, Camilla Viglio, Gianluca Folco, Francesco Rizzetto, Salvatore Zirpoli

**Affiliations:** 1 Pediatric Radiology Unit, “Vittore Buzzi” Children’s Hospital, Milan, Italy; 2 Postgraduate School in Radiodiagnostics, University of Milan, Milan, Italy; 3 Department of Pediatric Surgery, “Vittore Buzzi” Children’s Hospital, Milan, Italy

**Keywords:** Ovarian Neoplasms, Magnetic Resonance Imaging, Child, Neoplasms, Risk

## Abstract

**Objective:**

The purpose of our study was to test the applicability and implications of using the O-RADS system, which is developed and validated on adults, to review MRI of ovarian masses among pediatric patients.

**Materials and methods:**

We retrospectively reviewed consecutive MRI examinations from pediatric patients referred to imaging for suspected ovarian lesions, assessing them using the O-RADS framework. Malignancy frequencies among O-RADS classes were reviewed, and we appraised the potential for such approach to split patients into low (O-RADS 1, 2, and 3) and high risk (O-RADS 4 and 5). Multivariate analyses were conducted to review which clinical or imaging variables yielded a significant impact on malignancy, and a simplified reading framework was proposed accordingly.

**Results:**

109 female patients were included, with a median age of 13 years (IQR 11–15 years), 7 (7%) presenting with malignant lesions. Malignancy proportions were 0% (95% confidence Interval (CI) 0–35%) for the O-RADS 1 class, 0% (95% CI 0 − 5%) for the O-RADS 2 class, 0% (95% CI 0–14%) for the O-RADS 3 class, 50 (95% CI 1 − 99%) for the O-RADS 4 class, and 75% (95% CI 41–93%) for the O-RADS 5 class. The presence of peritoneal thickening or nodules (*p* < 0.001), lesion composition (*p* < 0.001), and absence of intralesional fat (*p* = 0.051) were individual predictors of malignancy, and the simplified reading framework proposed with such variables identified 11 likely malignant cases, detecting all 7 malignant lesions.

**Conclusion:**

The O-RADS system may be applied to MRI performed in the pediatric population for ovarian masses, and a simplified reading framework based on O-RADS could also prove useful in such a setting.

**Key Points:**

**Question** *The Ovarian-Adnexal Reporting and Data System (O-RADS) provides the risk of malignancy of ovarian masses among adults but has not been validated among pediatric patients*.

**Findings** Malignancy frequencies for O-RADS classes 1, 2, 3, and 4 were 0, 0, 50%, and 75%, indicating a good accuracy in lesion discrimination.

**Clinical relevance** *The Ovarian-Adnexal Reporting and Data System (O-RADS) can be effectively applied to MRI examinations in pediatric patients, enabling accurate classification of findings, with potential for score simplification in this age group*.

## Introduction

Pediatric ovarian malignancies are relatively rare, representing only a small percentage, close to 1%, of all pediatric malignancies [1]. Nevertheless, the likelihood of malignancy for ovarian masses found in childhood is nearly 10%, and as such, it is crucial to distinguish between malignant and benign ovarian masses to properly plan timely clinical management [2, 3]. Indeed, complete surgical resection is mandatory in case of malignant lesions, whereas benign lesions may be managed sparing healthy ovarian tissue. In this scenario, MRI plays a pivotal role in characterizing pediatric ovarian lesions and distinguishing between benign and malignant lesions when ultrasound results are inconclusive [4, 5]. Previous studies have shown that MRI reduces false positives, decreasing unnecessary invasive surgeries and preserving future fertility in pediatric patients. Moreover, the absence of ionizing radiation makes MRI especially suitable for children [6].

Concerning the use of MRI for ovarian masses, in 2021, the American College of Radiology proposed a system for the classification of ovarian/adnexal lesions, the Ovarian-Adnexal Reporting and Data System (O-RADS MRI), to support a standardized description of ovarian and adnexal masses at MRI, with indications for their management [6]. In particular, the O-RADS system identifies 6 distinct classes with increasing likelihood of malignancy [7]. Nevertheless, the O-RADS classification system in its present form is tailored to the adult population, which presents with different malignancy likelihood and cancer types than the pediatric population [8]. As such, it may not be assumed that results pertaining to O-RADS are applicable to pediatric patients and that management indications should be directly translated.

Hence, the purpose of our study was to review MRI examinations performed in patients referred to our pediatric radiology department for ovarian masses, evaluate the applicability of O-RADS in such a population, and explore the potential implications of its use.

## Materials and methods

### Study population

This study was approved by the local Ethics Committee of “Vittore Buzzi” Children’s Hospital (code: O-RADS MRI, approved on September 6th, 2023), and informed consent was waived due to its retrospective nature.

We conducted a search on our Picture Archiving and Communication System for all patients who were referred to MRI with a clinical question involving the word “ovary”, “ovarian”, “adnexa” or “adnexal”. Subsequently, all consecutive pelvic MRI examinations performed in pediatric patients for the suspicion of ovarian masses at our center since January 2012 were retrospectively included. We included patients with both symptomatic and asymptomatic adnexal masses, presenting with symptoms such as acute or chronic abdominal pain or abdominal distension. However, we excluded MRI examinations in cases where torsion compromised the evaluation of the adnexal lesion.

Additionally, we excluded incomplete MRI examinations, repeat MRI scans of the same ovary following the initial evaluation, MRI examinations of ovaries previously treated surgically or medically, MRI scans where the tubal origin of the abnormality was clearly identified, and MRI examinations from patients with ovarian lesions lacking either surgical confirmation or follow-up data.

### Image acquisition

All patients underwent MRI performed using a 1.5-T system (Philips Achieva Dual-D Stream, Philips) while lying supine using a 16-channel body coil. Imaging protocols included T2-weighted sequences, as well as pre- and post-contrast T1-weighted sequences (Dotarem, Guerbet, at 0.2 mL/kg) with and without fat suppression, also including T1 in and out of phase sequences, and diffusion-weighted imaging (DWI) with at least 3 b-values comprised between 0 and 1000.

### Image analysis

**Fig. 1:**
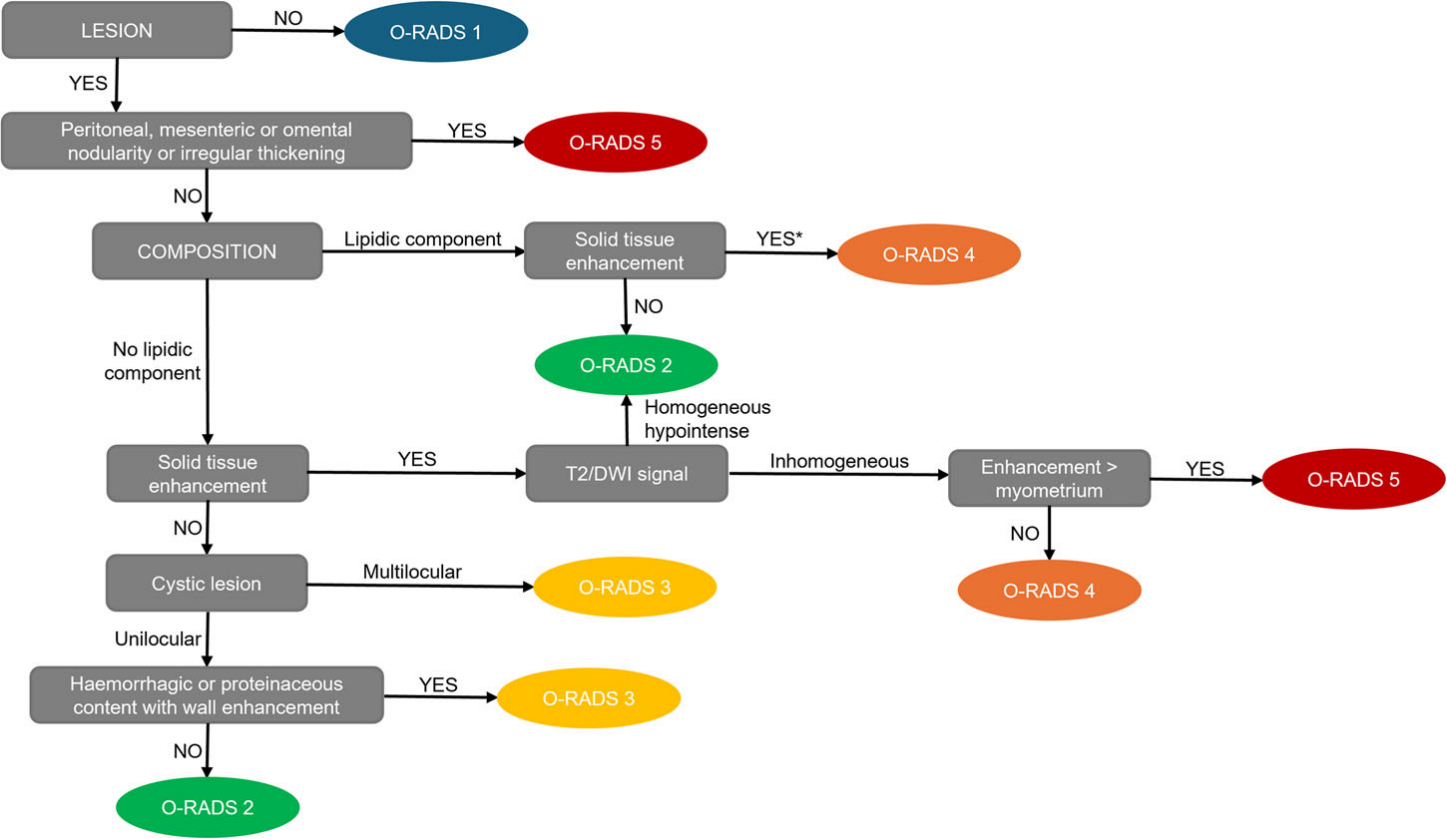
Image analysis flow-chart, derived from the Ovarian-adnexal Reporting And Data System (O-RADS) for magnetic resonance imaging [6]. *An enhancing component ≥ 8 cm was considered large, as per Janssen et al [9]

All MRI examinations were evaluated by a pediatric radiologist with 10 years of experience in pediatric pelvic MRI (A.M.M.), and by a resident in radiology with 2 years of experience in pediatric imaging (G.F.) who applied the O-RADS MRI criteria and assigned O-RADS classes based on several parameters. Figure 1 depicts the algorithm employed in the evaluation of ovarian masses.

**Fig. 2:**
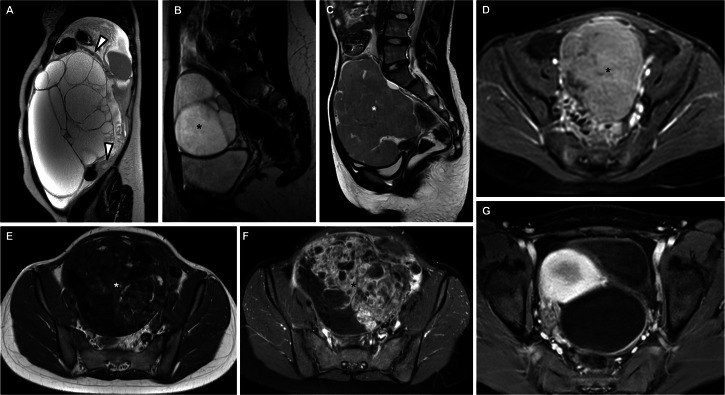
Examples of findings included in the Ovarian-adnexal Reporting And Data System (O-RADS) for magnetic resonance: peritoneal thickening with nodules (**A**, arrowheads) in a sagittal T2-weighted image acquired in a 14-year-old patient with histopathologically proven mucinous borderline ovarian tumor with foci of intraepithelial carcinoma; multilocular cystic lesion (**B**, asterisk) in a sagittal T2-weighted image acquired in a 14-year-old patient with serous cystadenoma; solid lesion consisting of at least 80% solid enhanced tissue in a sagittal T2-weighted image (**C**, asterisk) and axial, post-contrast T1-weighted image (**D**, asterisk) in a 10-year-old patient with histopathology-proven ovarian Burkitt’s lymphoma; fat-containing lesion with wide enhanced area greater than 8 cm in maximum diameter on an axial T1-weighted image (**E**, asterisk) and axial post-contrast T1-weighted image (**F**, asterisk) in a fifteen-year-old patient with a histopathology-proven malignant teratoma; cystic lesion (**G**) with non-fluid signal intensity on an axial, post-contrast T1-weighted image in a 12-year-old patient with a hemorrhagic cyst

First, we assessed the presence of an ovarian mass. If no mass was detected, the case was classified as O-RADS 1. For cases with a detectable mass, we evaluated the presence of peritoneal, mesenteric, or omental nodularity and/or irregular thickening, with or without ascites. Next, we noted the lipid content on T1-weighted images. If fat components were present, we measured enhancement areas and recorded if their maximum diameter was equal to or exceeded 8 cm for further analysis [9]. In the absence of lipid components, we assessed the presence of solid tissue according to the O-RADS MRI lexicon [6]. If solid tissue was present, we evaluated signal intensity on T2 (either homogeneously hypointense—with a uniform intensity equal to or below that of the iliopsoas muscle—or not), DWI (either homogenously not restricted or restricted, assessing both DWI images and ADC maps), and the degree of contrast enhancement (either hypo/isoenhancing or hyperenhancing relative to the myometrium). For cystic lesions without solid tissue, we assessed the following characteristics: unilocular or multilocular lesion; simple fluid content (fluid content with signal analogous to urine: hyperintense on T2-weighted imaging, hypointense on T1-weighted imaging) or non-simple fluid content, including endometriotic fluid (hypointense on T2-weighted imaging and hyperintense on T1-weighted imaging), hemorrhagic fluid (variable signal depending on its age), or proteinaceous fluid (variable signal on T2-weighted imaging and variably hypointense on T1-weighted imaging). Additionally, we assessed the presence of parietal enhancement in lesions with non-simple fluid. Figure 2 displays an example of findings included in the O-RADS classification.

### Statistical analysis

Continuous variables were tested for normality by visualizing graphs and the Shapiro-Wilk test. Normally distributed variables were reported as mean and standard deviation (SD), and analyzed with parametric statistics, whereas non-normally distributed variables were reported as median and interquartile range (IQR) and analyzed with nonparametric statistics. Categorical data were reported as counts and percentages, and 95% confidence intervals (95% CI) were considered when reporting malignancy frequencies. Then, lesions were grouped with regards to low (O-RADS 1, 2, and 3) or high (O-RADS 4 and 5) risk of malignancy, and malignancy proportions were calculated accordingly. Multivariate regression analyses were used to appraise which clinical and imaging variables were independent predictors of lesion malignancy, and a simplified version of the image analysis workflow was proposed and tested accordingly. Inter-reader agreement of O-RADS and simplified scores was assessed using Cohen’s linear weighted *κ*. The *p*-value threshold was set at *p* < 0.050, with *p* < 0.100 considered borderline significant [10].

## Results

### Study population

**Fig. 3:**
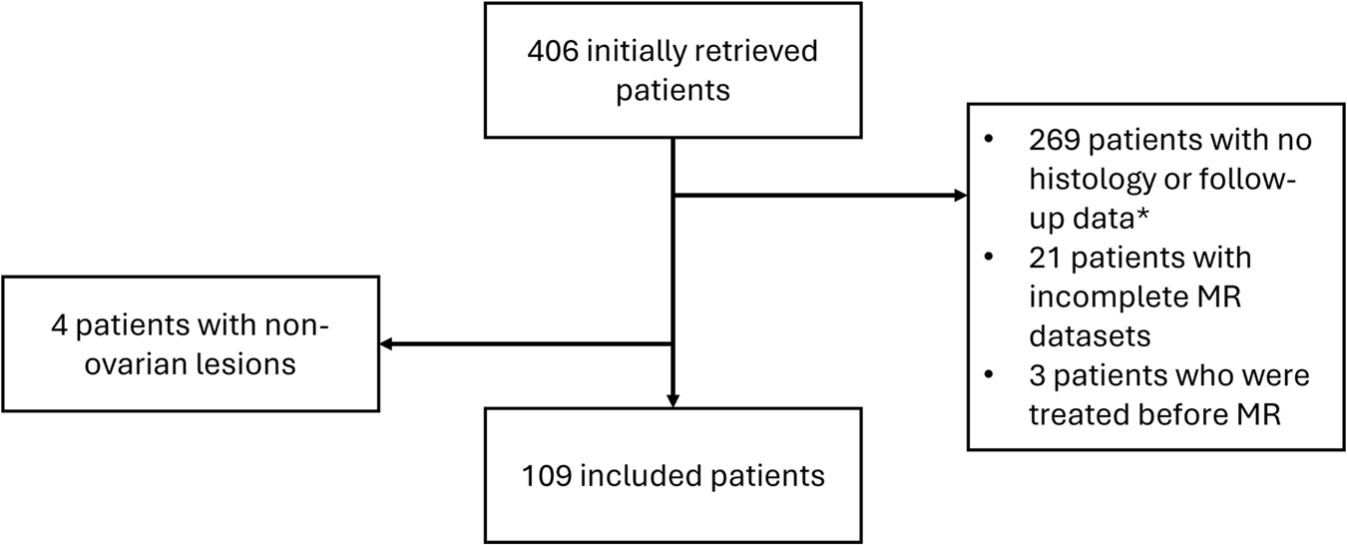
Flow-chart depicting the study selection process. *This does not include O-RADS 1 patients with no lesions, that do not warrant follow-up. MR: magnetic resonance

Overall, 109 female patients were included in our study, with a median age of 13 years (IQR 11–15 years). The study selection process is depicted in Fig. 3. Among them, 7/109 (6%) presented with malignant lesions, all of which were confirmed by histology: 2/7 (30%) patients were diagnosed with dysgerminoma, 1/7 (14%) with malignant teratoma, 1/7 (14%) with a mucinous borderline ovarian tumor with foci of intraepithelial carcinoma, 1/7 (14%) with a malignant granulosa cell tumor, 1/7 (14%) with a rhabdoid tumor, and 1/7 (14%) with ovarian Burkitt’s lymphoma. Among the 102/109 (93%) cases with benign findings, 68/102 (67%) had a pathology report, while 39 (343%) were classified as benign based on clinical and imaging follow-up findings. Patient information is reported in Table 1.

**Table 1:** Characteristics of the study population

	Study population
Age (years)	13 (11–15)
Lesion presence (*n*, %)	102 (94)
Size (mm)	62 (45–110)
O-RADS class	
2	68
3	23
4	2
5	9

*O-RADS* Ovarian-Adnexal Reporting And Data System

### Lesion assessment

**Fig. 4:**
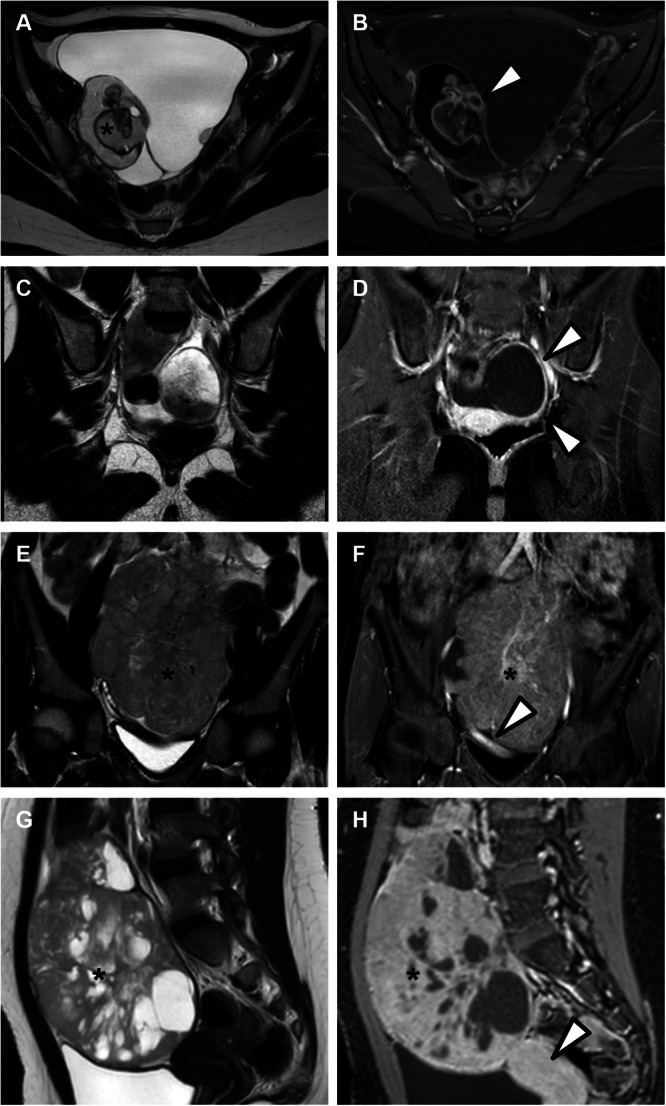
Examples of ovarian lesions classified into different O-RADS categories, shown on T2-weighted (left) and contrast-enhanced T1-weighted fat-suppressed (right) images, respectively. **A**, **B** O-RADS 2—Axial images of an 18-year-old patient with histopathology-proven mature teratoma reveal fat components (asterisk) within the lesion and a partially enhanced Rokitansky nodule (arrowhead). **C**, **D** O-RADS 3—coronal images of a 13-year-old patient with a symptomatic 6 cm unilocular cyst, histopathology-proven hemorrhagic cyst, showing heterogeneous T2 and T1 signal content with parietal enhancement (arrowhead). **E**, **F** O-RADS 4—coronal images of a 12-year-old patient with histopathology-proven ovarian dysgerminoma, showing a heterogeneous solid lesion with enhancement (asterisk) less intense than the uterine myometrium (arrowhead). **G**, **H** O-RADS 5—sagittal images of a 12-year-old patient with histopathology-proven ovarian malignant granulosa cell tumor, showing a solid and cystic lesion with enhancement (asterisk) more intense than the uterine myometrium (arrowhead)

Out of all patients, 102/109 (94%) presented with ovarian lesions, whereas 7/109 (6%) did not present with any lesion, and were thus classified as O-RADS 1. Among patients with lesions, 68/102 (67%) were classified as O-RADS 2, 23/102 (22%) as O-RADS 3, 2/102 (2%) as O-RADS 4 and 9/102 (9%) as O-RADS 5. Examples of O-RADS lesions are shown in Fig. 4. Average lesion size was 62 mm (IQR 45–110 mm), and 54/102 (53%) lesions were found in the right ovary, whereas 48 (47%) were observed in the left ovary. Examples of O-RADS lesions are shown in Fig. 4. Agreement between readers for O-RADS classes was substantial (*κ* = 0.766).

Among lesions classified as O-RADS 2 or 3, none were malignant, whereas among lesions classified as O-RADS 4, 1/2 (50%) was malignant, and among O-RADS 5 lesions, 6/9 (75%) were malignant. Thus, malignancy proportions were 0% (95% CI 0–35%) for the O-RADS 1 class, 0% (95% CI 0–5%) for the O-RADS 2 class, 0% (95% CI 0–14%) for the O-RADS 3 class, 50 (95% CI 1–99%) for the O-RADS 4 class, and 75% (95% CI 41–93%). Combining lesions classified as O-RADS 1, 2, or 3 as low risk and those classified as O-RADS 4 and 5 as high risk, the overall malignancy proportion was 0% (95% CI 0–3%) for low-risk lesions, and 64% (95% CI 35–85%) for high-risk lesions. Of note, benign lesions classified as O-RADS 4 or O-RADS 5 were all subject to histologic assessment and included a serous papillary cystadenoma (O-RADS 4), two fibromas, and an unspecified benign lesion (O-RADS 5). Information on MRI characteristics of included lesions alongside surgical intervention is reported in Table 2.

**Table 2:** MRI characteristics of included lesions for each Ovarian-adnexal Reporting And Data System (O-RADS) class, alongside information concerning surgical intervention

	O-RADS 2	O-RADS 3	O-RADS 4	O-RADS 5
	(*n* = 68)	(*n* = 23)	(*n* = 2)	(*n* = 9)
MRI characteristics (*n*, %)				
Peritoneal thickening or nodules	0	0	0	3 (33)
Fat component	46 (68)	0	0	2 (22)
Solid component enhancement (greater than 8 cm)	0	0	0	1 (11)
Homogenously T2 hypointense/restricted diffusion (solid)	0	0	0	0
Contrast enhancement compared to myometrium (solid/part solid)	0	0	2 (100)	4 (44)
Cystic lesions	63 (93)	23 (100)	0	0
Surgical intervention (*n*, %)	53 (78)	5 (22)	2 (100)	9 (100)
of which ovarian-sparing surgery	36 (68)	2 (40)	1 (50)	2 (22)
Malignant lesions (*n*, %)	0 (0)	0 (0)	1 (50)	7 (75)

### Determinants of malignancy

The clinical and imaging variables that yielded a significant, or borderline significant, impact on malignancy were the presence of peritoneal thickening or nodules (*p* < 0.001), lesion composition (*p* < 0.001) with purely cystic lesions being less malignant than lesions with solid components, and the absence of intralesional fat (*p* = 0.051), with fatty lesions displaying lower chance of malignancy. The other assessed clinical and imaging variables, namely age, maximum lesion dimension, presence of septa, and type of cystic fluid, did not yield a significant impact on lesion malignancy (*p* ≥ 0.102).

**Fig. 5:**
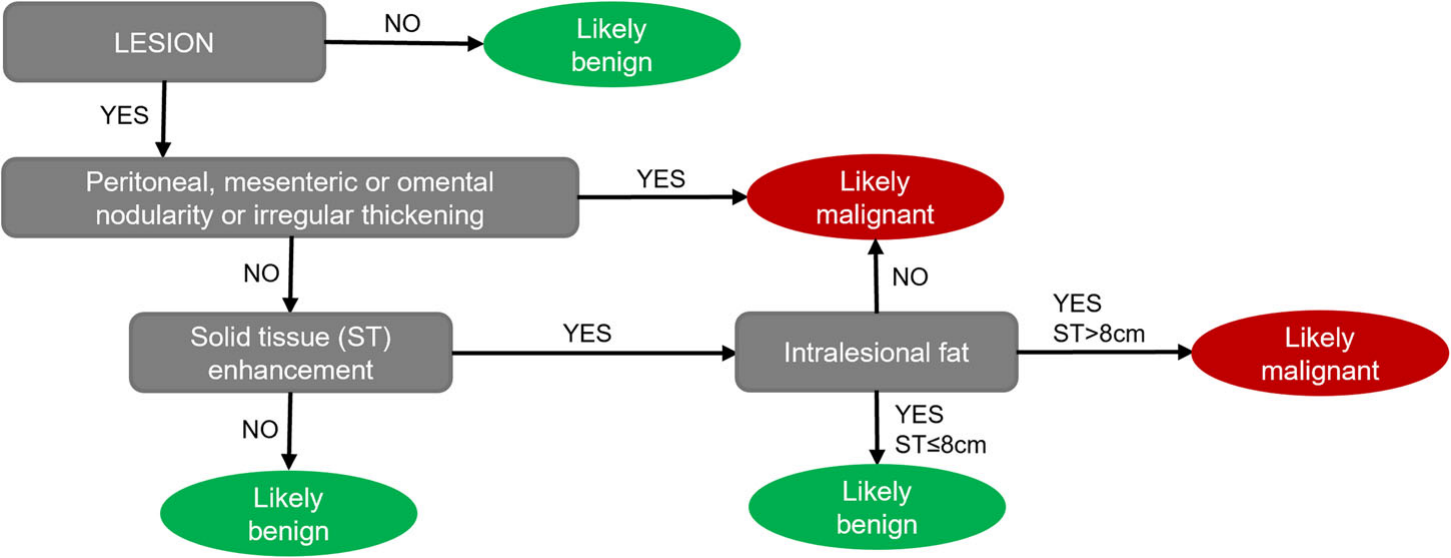
Simplified reading framework, derived from variables yielding a significant impact on lesion malignancy

The initial image analysis workflow was modified as per Fig. 5, leading to the classification of 11 (10%) patients as likely malignant, and 93 (90%) as likely benign. All 7 malignant cases were classified into the likely malignant group, while none were found in the likely benign group, leading to a malignancy proportion of 64% (95% CI 35–85%) for the former and of 0% (95% CI 0–4%) for the latter. Agreement for likely malignant and benign grouping was substantial (*κ* = 0.771).

## Discussion

The aim of our study was to review the applicability and implications of the use of the O-RADS classification system to MRI examinations from pediatric patients referred to imaging for ovarian masses. As opposed to ovarian masses in adult patients, which present with higher frequencies of malignancy [11], pediatric patients with ovarian masses have a chance of malignancy close to 10% [12], as confirmed by our study population, with an overall malignancy rate of 6% (95% CI 2–12%). This difference in malignancy rates is due to the fact that the histology of ovarian tumors differs widely between pediatric patients and adults, with benign cystic teratoma being the most common neoplasm in children, and germ cell tumors being the most frequent malignancy [13, 14]. Furthermore, such findings indicate that while the O-RADS system is applicable to pediatric patients, and leads to reasonable results, being characterized by growing rates of malignancy for each increasing class, it may not represent the best fit for mass classification in such population, as the first three classes all present with virtually no malignancy. Indeed, simplifying the classic O-RADS system by merging classes 1, 2, and 3 and classes 4 and 5 as high risk of malignancy, as also suggested by previous studies conducted on adults [15], may lead to a more straightforward classification of ovarian masses.

As determinants of lesion nature, we observed that peritoneal thickening or nodules were an independent predictor of malignancy, which may appear straightforward, as only malignant lesions metastasize to the peritoneum [16]. Furthermore, simple cystic lesions are most often related to functional ovarian cysts, which are commonly observed in children and adolescents, leading to a purely cystic composition being indicative of lesion benignity [17]. Similarly, the absence of intralesional fat is related to lesion benignity, as it is a most common finding in patients presenting with mature teratomas [14]. A significant number of patients exhibited lesions containing fatty tissue. All these fatty lesions were initially classified as benign except for one. Histological examination confirmed the benign nature of teratomas and a mucinous cystadenoma arising from a mature cystic teratoma. The sole lesion, initially classified as malignant due to its large (> 8cm) solid-enhancing area, was subsequently confirmed to be a malignant teratoma at histological analysis. None of the other clinical and imaging variables were shown to be independent predictors of lesion malignity, despite their important role in the adult classification, possibly due to the different histology. Consequently, the proposed simplification of the O-RADS classification flow-chart may present a feasible option, as it still allows the classification of all malignant lesions as high risk without missing any while still keeping false positives to a minimum. This approach would also allow a quicker and easier MRI classification, which could possibly be performed with high reproducibility even by less experienced readers.

A study by Wang et al [18] tested the O-RADS system for ultrasound evaluation in pediatric patients, concluding that using an O-RADS score greater than 3 as a cutoff achieved good diagnostic performance in identifying malignant ovarian masses. These findings align with our study, suggesting that simplifying the O-RADS system used for adults may be feasible for pediatric patients, leading from a 5-class system to a binomial outcome.

Hence, patients with benign findings may be directed to follow-up or observation, with less invasive surgery being an option in case of voluminous, symptomatic masses or subsequent ovarian torsion [18]. Conversely, high-risk lesions, with an increased likelihood of malignancy, could be referred to oncologic pathways for proper diagnosis and management, with a relatively small percentage of false positive cases [19]. As such, similarly to recommendations provided by the European Society of Urogenital Radiology for adult patients [20], MRI with the aid of the O-RADS framework could be used in pediatric patients with lesions that are indeterminate at ultrasound, as with a high diagnostic performance in such group [5], to plan appropriate clinical management.

Our study presents some limitations. First, our study population is retrospective and counts only a few patients with malignant ovarian masses, and some types of lesions that may not be correctly classified by the O-RADS system may not be represented in our study group. However, the most common ovarian malignancy in childhood, namely germ cell tumors which represent about 80% of malignancies in such group [21], are present in our study population and are properly recognized as high risk by the application of O-RADS on MRI scans, leading to the supposition that such findings could be confirmed by studies conducted on multi-centric populations, with higher numbers of malignant cases. Moreover, despite literature recommending dynamic MRI acquisitions with gadolinium-based contrast agents [5], such datasets were unavailable for our entire study population which includes examinations from as early as 2012. Thus, fewer cases might have been wrongly labeled as malignant or high risk if such acquisitions were available; for instance, two fibromas were classified as O-RADS 5 based only on delayed-phase imaging. Furthermore, throughout the entire period of dataset inclusion for the present study clinical practice has changed, also in relation to the review and renewal of related guidelines. The fact that clinical practice changed during such time may represent a study limitation, as differences in imaging protocols and quality may arise, acting as potential confounders. Nevertheless, we do believe that such changes have not yielded a significant impact on the variables included in the O-RADS framework, which were evaluated in the present work and are assessable over a varied range of study protocols and clinical settings.

In conclusion, the O-RADS system may be applied to MRI performed in the pediatric population for ovarian masses. Moreover, a simplification of such system, only relying on the evaluation of few variables, may be feasible, and further studies are warranted to validate such hypothesis.

## Abbreviations

95% CI: 95% Confidence interval
DWI: Diffusion-weighted imaging
O-RADS: Ovarian-Adnexal Reporting and Data System
